# The mediating role of psychological inflexibility on internalized stigma and patient outcomes in a sample of adults with inflammatory bowel disease

**DOI:** 10.1093/ecco-jcc/jjaf055

**Published:** 2025-04-01

**Authors:** Darren P Reynolds, Trudie Chalder, Claire Henderson

**Affiliations:** King’s College London, Institute of Psychiatry Psychology & Neuroscience, Department of Psychology, London, United Kingdom; King’s College London, School of Population Health and Environmental Sciences, London, United Kingdom; King’s College London, Department of Psychological Medicine, Department of Psychological Medicine, London, United Kingdom

**Keywords:** inflammatory bowel disease, psychological inflexibility, internalized stigma, health-related quality of life, psychological distress

## Abstract

**Background:**

This study examined the relationship between psychological inflexibility, internalized stigma, and patient outcomes in adults with inflammatory bowel disease (IBD). It aimed to explore if psychological inflexibility mediated the relationship between internalized stigma and patient outcomes.

**Methods:**

Three hundred and eighty-two participants with IBD took part in a cross-sectional quantitative study conducted via an online survey from May to December 2020. Participants completed questionnaires that assessed psychological inflexibility, committed action, internalized stigma related to IBD, psychological distress, IBD self-efficacy, self-concealment, beliefs about emotions, and fatigue. Participants also completed a sociodemographic and clinical questionnaire in addition to a bespoke Covid-19 questionnaire. Pearson’s correlations and exploratory simple mediation analyses were used to examine relationships between variables and the mediating effect of psychological inflexibility.

**Results:**

40.5% of participants experienced internalized stigma. Higher psychological inflexibility was associated with higher internalized stigma, lower committed action, poorer health-related quality of life, lower IBD self-efficacy, higher self-concealment, higher fatigue, and more negative beliefs about emotions. Psychological inflexibility partially mediated the relationship between internalized stigma and several patient outcomes (psychological distress, IBD health-related quality of life, IBD self-efficacy, and self-concealment) and completely mediated the relationship between internalized stigma and fatigue.

**Conclusion:**

Psychological inflexibility significantly impacts the quality of life in individuals with internalized stigma related to IBD and mediates the relationship between internalized stigma and patient outcomes. Increasing psychological flexibility in adults with IBD may reduce distress and enhance quality of life. Longitudinal studies and trials of psychological interventions targeting psychological flexibility warrant exploration.

## 1. Introduction

### 1.1. Inflammatory bowel disease

Inflammatory bowel disease (IBD) encompasses chronic gastrointestinal conditions, primarily Crohn’s disease (CD) and ulcerative colitis (UC). IBD is most prevalent in Caucasian populations, affecting 0.3–0.5% of individuals in Western countries,^1–3^ with global prevalence rising.^2,4^ In Europe and the United States, UC affects 120–200 per 100 000 people, while CD affects 50–200 per 100,000.^3^ Common symptoms include abdominal pain, diarrhea, anemia, and fatigue.^5^ Bowel urgency is a major concern,^6–10^ with up to 74% of patients experiencing some degree of fecal incontinence, and 9% reporting regular incontinence.^11^

IBD significantly affects occupational,^12–14^ psychosocial,^15–17^ and health-related quality of life,^12,18,19^ particularly during flare-ups.^20,21^ Psychological distress, linked to fatigue, social isolation, bowel urgency, and body image concerns (eg, surgical scarring or ostomy use),^17,22–28^ is more prevalent in individuals with IBD. Depression and anxiety rates are higher than in the general population,^27,29–31^ and distress can exacerbate symptoms,^32^ especially during active disease phases,^27^ contributing to increased healthcare costs.^33^

### 1.2. Stigma and “Internalised Stigma”

Stigma occurs when societal norms devalue individuals based on health conditions, identities, or backgrounds, leading to social and psychological distress such as shame and a spoiled identity.^34–36^ Initially studied as a personal attribute,^37^ stigma is now understood as shaped by discrimination and power structures.^38^

**Figure 1.**
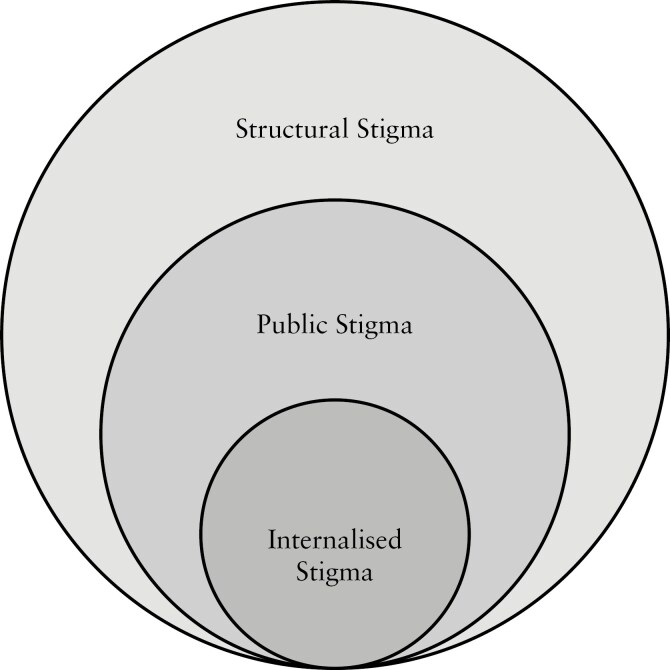
Stigma as a multi-level construct consisting of three interacting levels: structural stigma, public stigma, and internalized stigma.

Stigma operates on three levels: structural, public, and internalized (see Figure 1).^39^ Structural stigma restricts rights through policies and institutional practices.^40–42^ Public stigma reinforces stereotypes and discrimination at the societal level.^41,43^ Internalized stigma, or self-stigma, occurs when individuals adopt these negative beliefs as their own.^44–46^

### 1.3. Internalized stigma and IBD

IBD symptoms, including bowel urgency, weight loss, and surgical interventions, can contribute to stigma,^3^ exacerbated by social norms surrounding bowel control.^47,48^ Research indicates that bowel-related issues are a widespread cultural taboo.^49,50^ Historically, IBD was seen as psychosomatic, further stigmatizing the condition.^51^ Treatment primarily focuses on symptom relief, with surgery often necessary for severe cases.^52^ Surgical scars and stoma bags can impact body image and intimacy, leading to stigma.^53,54^

Reviews confirm that IBD is susceptible to stigma due to misconceptions, socially unacceptable symptoms, and concealability.^55,56^ Research often focuses on felt-stigma, where individuals perceive stigma from others, leading to condition concealment.^57,58^ Felt-stigma is linked to poorer outcomes such as lower quality of life, increased anxiety and depression, and reduced self-efficacy and self-esteem.^50^ While coping strategies may buffer felt-stigma, internalized stigma can have more severe effects.^55^

Research on internalized stigma in IBD is limited. One study found that IBD patients with a colostomy associated their condition with feelings of disgust and shame.^54^ Another study linked higher health-related self-blame to poorer outcomes and coping strategies.^59^ A study specifically investigating internalized stigma in IBD found that 36% of patients experienced internalized stigma, with associated reductions in quality of life, health competence, psychological functioning, and increased healthcare utilization.^60^ More research is needed to understand how internalized stigma affects self-management and patient outcomes.^55,56^

### 1.4. Psychological flexibility and psychological inflexibility

Psychological flexibility—the ability to be open to distressing internal experiences (eg, thoughts, body sensations, and feelings) while still engaging in valued actions—is central to Acceptance and Commitment Therapy (ACT^61^; see Figure S1 in the Supplementary Material), a third-wave cognitive behavioral therapy. Psychological flexibility is considered a key component of psychological health^62,63^ and helps individuals adapt their behavior to align with values, even under fluctuating situational demands.^62^

For individuals with IBD, psychological flexibility may mitigate internalized stigma. Those with greater awareness of their internal experiences may be more aware of and open to stigmatizing thoughts, enabling them to engage in valued actions despite these thoughts. Consequently, higher levels of internalized IBD-related stigma would be expected to correlate with greater psychological inflexibility.

### 1.5. Rationale and purpose of the current study

Individuals with IBD experience stigma related to their condition,^55,56^ which can be internalized and lead to poorer quality of life.^60^ Given the theoretical importance of psychological inflexibility in internalized stigma, this study explored the relationship between psychological inflexibility, internalized stigma, and patient outcomes (eg, psychological distress, health-related quality of life, self-efficacy, self-concealment, beliefs about emotions, and fatigue) in adults with IBD.

The study aimed to (a) assess levels of internalized stigma, psychological inflexibility, and key patient outcomes (health-related quality of life, psychological distress, IBD self-efficacy, self-concealment beliefs about emotions, and fatigue); (b) examine relationships among these variables; and (c) investigate whether psychological inflexibility mediates the relationship between internalized stigma and key patient outcomes. Additionally, exploratory mediation analyses examined whether psychological inflexibility mediates the relationship between public stigma and internalized stigma.

No previous research has examined these relationships in an adult IBD population. This study aims to fill this gap, enhancing understanding of the psychological factors that contribute to internalized stigma and distress. Findings could inform psychological interventions to improve the quality of life for individuals with IBD experiencing internalized stigma.

Based on prior research, we hypothesized that individuals with greater internalized stigma would exhibit higher psychological inflexibility, leading to poorer patient outcomes. We also expected internalized stigma and psychological inflexibility to be linked to increased self-concealment and beliefs that negative emotions should not be expressed. Lastly, we predicted that psychological inflexibility would mediate the relationships between internalized stigma and patient outcomes, as well as mediate the relationship between public stigma and internalized stigma by demonstrating statistically significant indirect paths in a series of simple mediation analyses.

## 2. Materials and methods

### 2.1. Study design and procedure

This study used a cross-sectional quantitative questionnaire design, collecting data from May to December 2020 via convenience sampling. Approved by the King’s College London PNM Research Ethics Panel (REC reference: LRS-19/20-18288), the online questionnaire was hosted on SurveyMonkey (SurveyMonkey Inc., San Mateo, California, USA; http://www.surveymonkey.com) and included 11 outcome measures, taking approximately 25 minutes to complete. Participants could use a computer, tablet, or smartphone, with cookies enabled to prevent multiple submissions from the same device. As an incentive, participants could enter a prize draw for one of ten £15 Amazon eVouchers.

The study was promoted on social media (Twitter, Instagram, Facebook) and through high-profile IBD charities like Crohn’s & Colitis UK, IBD Relief, forCrohn’s, King’s College London’s Crohn’s and Colitis Society, and IBD UK. It was also advertised on Reddit forums (/r/IBD, /r/CrohnsDisease, /r/ulcerativecolitis) and circulated to King’s College London staff and students via a research newsletter.

### 2.2. Participants

A total of 382 (*n* = 302 female) individuals with IBD participated. Inclusion criteria for participants were (1) a confirmed diagnosis of IBD, (2) aged 18 years and above, (3) the ability to read, understand, and respond to questionnaires that were only available in English, and (4) agreement to participate in the study. Exclusion criteria consisted of (1) refusal to participate, (2) not having a confirmed diagnosis of IBD, and (3) being unable to understand or complete the questionnaire measures. Participants read an information sheet and provided informed consent before answering the questionnaire.

### 2.3. Study measures

To meet the aims of the current study, the online questionnaire consisted of 11 separate study measures which were as follows:

#### 2.3.1. Socio-demographic and IBD status questionnaire

The research team designed a bespoke questionnaire (see File S1 in supplementary material) to collect demographic data (eg, age, gender, ethnicity, education, relationship status) and IBD clinical characteristics (eg, diagnosis, time since diagnosis, symptom severity over past 3 months, flare frequency, steroid dependence, ostomy presence, hospital admissions over the past year related to IBD diagnosis).

#### 2.3.2. Acceptance and action questionnaire II (AAQ-II)

The AAQ-II^64^ is a seven-item self-report measure of psychological inflexibility, assessing acceptance of emotional experiences. Respondents rate statements on a 7-point Likert scale (1 = “Never true” to 7 = “Always true”). Scores range from 7 to 49, with higher scores indicating greater psychological inflexibility. The AAQ-II has good construct validity and reliability (α = 0.84).^64^ Developed from the Acceptance and Action Questionnaire (AAQ),^65^ the AAQ-II shows a strong correlation (*r* = 0.97) with the original and better psychometric consistency, making it widely used.^66^ In this study, internal consistency was α = 0.92.

#### 2.3.3. Committed action questionnaire (CAQ-8)

The CAQ-8^67^ is an eight-item self-report measure of valued goal-directed action, adapted from the original 18-item CAQ.^68^ Each item is a first-person statement rated on a 7-point Likert scale (0 =  “Never true” to 6 =  “Always true”). Scores range from 0 to 48, with higher scores indicating higher valued goal-directed action. The CAQ-8 shows strong positive correlations with the CAQ, indicating good construct validity,^67^ and has good reliability with internal consistency ranging from α = 0.70 to 0.87.^67,69,70^ In this study, the internal consistency was α = 0.85.

#### 2.3.4. Internalized stigma scale for mental illness (ISMI-29)

The ISMI-29^71^ is a 29-item self-report measure assessing internalized stigma for mental illness.^72^ Respondents rate agreement with statements on a 4-point Likert scale. It has five subscales: Alienation (6 items assessing feelings of being devalued), Stereotype Endorsement (7 items assessing agreement with negative stereotypes), Discrimination Experience (5 items assessing perceived mistreatment), Social Withdrawal (6 items assessing avoidance of others), and Stigma Resistance (5 items assessing ability to deflect stigma).

In the current study, an adapted version of the ISMI-29 for IBD^60^ was used. Each item on the adapted version replaces the words “mental illness” with “IBD” and the item “People with mental illness tend to be violent” is modified to “People with IBD tend to be dirty.” The ISMI-29 shows good reliability and validity across cultures^72^ and when modified for long-term physical health conditions.^73^ Subscale scores range from 1.00 to 4.00, with higher scores indicating higher internalized stigma. The Stigma Resistance subscale is poorly correlated with others and lacks internal consistency,^74^ so it is excluded from the total score.^71,72,75^ The modified total score is calculated by summing 24 items and dividing by 24, yielding a score between 1.00 and 4.00. The modified total score has been utilized in a number of studies^60,76,77^ and has demonstrated excellent internal consistency.^75^

The study used a four-category interpretation for ISMI scores: 1.00–2.00 (minimal to no stigma), 2.01–2.50 (mild stigma), 2.51–3.00 (moderate stigma), and 3.01–4.00 (severe stigma).^78^ Stigma resistance was calculated separately. In the current study, the ISMI-29 total score had an internal consistency of α = 0.89. Subscale alphas were α = 0.85 (Alienation), α = 0.77 (Stereotype Endorsement), α = 0.83 (Discrimination Experience), α = 0.88 (Social Withdrawal), and α = 0.59 (Stigma Resistance).

#### 2.3.5. Depression anxiety and stress scales -21 (DASS-21)

The DASS-21^79^ is a 21-item self-report measure of psychological distress, derived from the 42-item DASS.^80^ Each item is rated on a 4-point Likert scale based on the past week’s experiences. The DASS-21 includes seven items each for depression, anxiety, and stress. Subscale scores (0–42) are calculated by summing relevant items and multiplying by 2.^80,81^ Higher scores indicate greater psychological distress. Total DASS-21 scores are calculated by summing the three subscale scores producing a single score of between 0 and 126 with higher scores indicative of greater psychological distress and hence poorer psychological functioning. The DASS-21 has good reliability and validity.^79,81^ In the current study, the internal consistency estimate for the DASS-21 total score was α = 0.96. Subscale alphas were α = 0.91 (Depression), α = 0.89 (Anxiety), and α = 0.92 (Stress).

#### 2.3.6. Crohn’s and ulcerative colitis questionnaire-8 (CUCQ-8)

The CUCQ-8^82^ is an 8-item self-report measure for health-related quality of life in individuals with IBD, derived from the 32-item CUCQ-32.^82^ It evolved from the UK-Inflammatory Bowel Disease Questionnaire (UK-IBDQ),^83^ an adaptation of the original IBDQ.^84^ The CUCQ-8 is less time consuming and does not require a license fee, making it suitable for clinical practice in both stable and acute IBD cases.^85^ It is the only IBD quality of life index used as a primary outcome in a randomized controlled trial.^86^ The CUCQ-8 assesses participants’ health-related quality of life over the past two weeks through six closed-ended questions (scored 0-14) and two items on a 4-point Likert scale. Each item on the CUCQ-8 is scored between 0 and 1 with raw scores on the closed-ended questions being divided by 14, and raw scores on the Likert scale items being divided by 3. As such, the total CUCQ-8 scores can range from 0 to 8, with lower scores indicating better quality of life. The internal consistency estimate for the CUCQ-8 in the current study was α = 0.77.

#### 2.3.7. IBD self-efficacy scale (IBDSES)

The IBDSES^87^ is a 29-item self-report measure assessing self-efficacy in managing IBD over the past 2 weeks. It covers managing stress and emotions (9 items), medical care (8 items), symptoms and disease (7 items), and remission (5 items). Responses use a 10-point Likert scale (1 = “Not sure at all” to 10 = “Totally sure”). Scores range from 29 to 290, with higher scores indicating higher self-efficacy. The IBDSES has excellent reliability (Cronbach’s α = 0.96) and good construct, concurrent validity, and test–retest reliability.^87^ The internal consistency estimate in the current study was α = 0.95.

#### 2.3.8. Chalder fatigue scale (CFS)

The CFS^88^ is a widely used self-report measure of fatigue. It assesses fatigue over the past month using a 4-point Likert scale (0 = “Less than usual” to 3 = “Much more than usual”). The first seven items address physical fatigue (eg, “Do you feel weak?”), and the last four address mental fatigue (eg, “Do you have difficulties concentrating?”). Scores range from 0 to 33, with higher scores indicating greater fatigue. The CFS has demonstrated good convergent validity^89^ and internal consistency (α = 0.86–0.92).^90–92^ It has been used in various health conditions, including CFS/ME,^93,94^ multiple sclerosis,^95^ cancer,^96^ and within the general population,^97^ showing reliable specificity and sensitivity.^92^ In this study, the internal consistency was α = 0.91.

#### 2.3.9. Beliefs about emotions scale (BES)

The BES^98^ is a 12-item self-report measure assessing beliefs about the unacceptability of expressing and experiencing negative emotions. Respondents rate agreement with statements on a 7-point Likert scale (0 = “Totally disagree” to 6 = “Totally agree”). Six items address the expression of negative emotions and six items address the experience of negative emotions. Scores range from 0 to 72, with higher scores indicating stronger beliefs that the expression and experience of emotions are unacceptable. The BES has good internal consistency (α = 0.96), sensitivity to change, and construct validity.^98^ In this study, internal consistency was α = 0.92.

#### 2.3.10. Self-concealment scale (SCS)

The SCS^99^ is a 10-item self-report measure assessing the tendency to conceal personal information due to fear of judgment or embarrassment. Each item is a first-person statement rated on a 5-point Likert scale (1 = “Strongly disagree” to 5 = “Strongly agree”). Scores range from 10 to 50, with higher scores indicating increased self-concealment. The SCS has shown good validity, internal consistency (α = 0.83 to 0.87), and test–retest reliability (*r* = 0.74–0.81).^99,100^ In this study, the internal consistency estimate was α = 0.90.

#### 2.3.11. Covid-19 questionnaire

The coronavirus disease 2019 (Covid-19) caused by SARS-CoV-2,^101^ led to a global pandemic declared by the World Health Organisation in March 2020.^102^ The Covid-19 Questionnaire, designed by researchers (see File S2 in the Supplementary Material for a copy of the questionnaire), aimed to collect data on participants’ experiences during the pandemic. The study began in May 2020, during widespread lockdowns in countries like the United Kingdom,^103^ restricting non-essential activities to curb the virus spread.^104^ The pandemic caused significant social and economic disruption, impacting mental health.^105^ UK statistics suggested that 32% of individuals in the United Kingdom reported high levels of anxiety, 43% reported a decrease in their general well-being, and 23% reported feeling lonely.^106^

The questionnaire aimed to identify differences in pandemic-related experiences that could act as covariates, considering the link between psychological distress and IBD symptoms,^32^ and the comorbidity of IBD and mental health issues,^107,108^ influenced by the gut-brain axis.^109–111^ Higher psychological distress, linked to increased internalized stigma within IBD,^60^ was expected among those most affected by the pandemic, potentially leading to poorer outcomes like reduced quality of life and increased fatigue.

Participants were asked about Covid-19 diagnosis, symptoms, life events due to the pandemic (eg, job loss, death of a loved one, financial decline), self-isolation status, risk perception, worry about Covid-19, living situation, and frequency of leaving home.

### 2.4. Data analysis

All quantitative data was analyzed using Microsoft Windows Excel for Office 365 (Microsoft, Redmond, WA, USA) and IBM SPSS Statistics for Windows, Version 27 (IBM, Armonk, NY, USA). Mean values and standard deviations were calculated for all outcome measures and demographic data, and data from continuous and interval measures were checked for normality. Cronbach’s α coefficient measured the reliability of all outcome measures in the IBD sample.

Participants were classified as having “low” or “high” psychological inflexibility based on their AAQ-II scores compared to the mean AAQ-II score. Means and standard errors for all study variables were calculated for both low and high psychological inflexibility groups, and independent *t*-tests compared differences between groups on all study variables.

Internalized stigma was reported using the ISMI-29 total scores, classified into four categories: minimal to no stigma, mild stigma, moderate stigma, and severe stigma.^78^ Means and standard errors for all study variables were calculated for these groups, and one-way ANOVAs with post-hoc Tukey tests compared the variables. Descriptive statistics analyzed data from the Covid-19 questionnaire.

Correlation analyses assessed relationships between study variables. Pearson’s correlations were computed, with values classified as weak, moderate, or strong.^112^ Point-biserial correlations compared study outcome variables to dichotomous demographic variables. Correlations were also run between study variables, demographic information, and relevant Covid-19 information to check for associations.

In a mediation model an independent variable (X) has an indirect effect (‘c) on a dependent variable (Y) via a third mediating variable (M)^113^ (see Figure S2 in the Supplementary Material). Mediation analyses examined psychological inflexibility as a mediator between internalized stigma and patient outcomes. We also examined whether psychological inflexibility would mediate an association between public stigma (also referred to as perceived stigma or enacted stigma as measured by the Discrimination Experience subscale of the ISMI-29^114,115^) and internalized stigma. The PROCESS^©^ computation macro plugin (Version 3.5, Model 4^116^;) for IBM SPSS was used to conduct these analyses, generating point estimates and bias-corrected confidence intervals (BC CIs) through bootstrapping. Mediation was deemed significant if BC CIs did not include zero.^117^ A 95% BC CI bootstrap resampling procedure (5000 samples) was used. A minimum sample size of *n* = 196 was suggested to provide at least 80% power to detect partial mediation at the 5% significance level.^118^ Diagrams generated by PROCESS^©^ visualized the mediation models with standardized coefficients for each of the *a*, *b*, *c*, and ‘*c* paths. A conventional alpha of 0.05 was set for all statistical tests.

## 3. Results

### 3.1. Response and completion rate

Out of 567 participants who accessed the study, 508 met the inclusion criteria, and 470 provided their demographic data. Of these 470, a total of 382 (81.3% completion rate) completed all 11 questionnaires and as such formed the final sample of the study. Given the large sample size and a small proportion of non-completers, listwise deletion (complete case analysis) was used for the current study, meaning that if a respondent did not complete the entire online survey then their data was not used. Several *t*-tests and Fisher’s exact tests were run to compare available data for those who had a complete response set (completers) to those who had a partial response set (non-completers). The only significant difference was found in regards to age, *t*(468) = 2.549, *P* = .011, with completers being older (*M* = 32.86, *SD* = 12.48) than non-completers (*M* = 29.17, *SD* = 11.17).

### 3.2. Demographic and clinical characteristics of the study sample

A full breakdown of the demographic characteristics of the sample can be seen in Table 1. The sample consisted of 382 participants, mostly female (79.1%), aged 18–77 (*M* =* *32.86, *SD* = 12.48). The majority of participants were of White/Caucasian ethnicity (87.4%). Most were in a relationship (56.0%) and had a certificate of higher education or higher (66.2%). Employment status was 64.0% employed. Sources of participant recruitment included IBD charities (26.4%), social media (56.8%), a King’s College London Participant Recruitment Research Newsletter (2.4%), and word of mouth (4.7%). The remainder of the participants were unsure about where they had heard about the study or did not report this information (9.7%).

**Table 1.** Demographic characteristics of study sample.

Variable	*n* (%)
***Gender:***	
Male	77 (20.2%)
Female	302 (79.1%)
Other (eg, Trans, Non-binary)	3 (0.8%)
***Age:***	*M* = 32.86
	*SD* = 12.48
***Ethnicity:***	
White/Caucasian:	334 (87.4%)
Mixed/Multiple Ethnic Groups:	21 (5.5%)
Asian/British Asian:	18 (4.7%)
Black/African/Caribbean/Black British:	4 (1.1%)
Other Ethnic Group:	5 (1.3%)
***Relationship Status:***	
Single	160 (41.9%)
Married	108 (28.3%)
Widowed	2 (0.5%)
Separated	1 (0.3%)
Divorced	2 (0.5%)
Long-term relationship	45 (11.8%)
Co-habiting	60 (15.7%)
Civil partnership	1 (0.3%)
Prefer not to say	3 (0.8%)
***Highest Level of Education Received:***	
* No qualifications*	12 (3.1%)
* Foundation diploma/GCSE (grades D-G)/NVQ Level 1*	12 (3.1%)
Higher diploma/GCSE (grades A* - C)/NVQ Level 2	32 (8.4%)
* Advanced diploma/A Level/BTEC National/NVQ Level 3*	73 (19.1%)
* Certificate of Higher Education/BTEC Professional/NVQ Level 4*	35 (9.2%)
* Bachelor’s degree (with honors)*	129 (33.8%)
* Postgraduate certificate or diploma/Master’s degree*	79 (20.7%)
* Doctorate degree*	10 (2.6%)
***Employment Status:***	
Employed full-time	168 (44.0%)
Employed part-time	54 (14.1%)
Self-employed	22 (5.8%)
Unemployed (currently looking for work)	23 (6.0%)
Unemployed (not currently looking for work)	12 (3.1%)
Student	70 (18.3%)
Retired	12 (3.1%)
Homemaker	11 (2.9%)
Unable to work	10 (2.6%)
***Source of Participant Recruitment:***	
IBD Charities	101 (26.4%)
Social Media (e.g. Twitter, Facebook & Instagram)	217 (56.8%)
KCL Participant Research Newsletter	9 (2.4%)
Word of mouth	18 (4.7%)
Unsure/Didn’t say	37 (9.7%)

A full breakdown of the clinical characteristics of the sample can be seen in Table 2. Diagnoses of participants included Crohn’s disease (45.0%), ulcerative colitis (42.9%), microscopic colitis (0.3%), and IBD unclassified (11.8%). Time since diagnosis (in months) ranged from 1 to 756 months or 63 years (*M* = 97.78, *SD* = 102.18). The majority of the sample was diagnosed more than 2 years ago (71.7%), and the remainder were diagnosed within the last two years (26.7%) or reported that they were unsure or could not remember when they were diagnosed (1.6%). IBD symptom severity varied; 12% of participants described symptom severity as “not active,” 33.2% as “minimally active,” 15.4% as “moderately active,” and 6.8% as “severely active” over the past 3-month period. 10.2% of the sample had an ostomy (eg, colostomy, ileostomy), and 14.4% were currently prescribed steroids as part of their IBD treatment.

**Table 2.** Clinical characteristics of study sample.

Variable	*n* (%)
***Diagnosis:***	
Crohn’s Disease	172 (45.0%)
Ulcerative Colitis	164 (42.9%)
Microscopic Colitis	1 (0.3%)
IBD Unclassified	45 (11.8%)
***Time period since diagnosis:***	
Within the last 2 years	102 (26.7%)
More than 2 years ago	274 (71.7%)
Unsure/can’t remember	6 (1.6%)
***Exact time since diagnosis (months):***	*M* = 97.78
	*SD* = 102.18
***IBD symptom severity over the past 3 months:***	
Not active	46 (12.0%)
Minimally active	127 (33.2%)
Mildly active	124 (32.5%)
Moderately active	59 (15.4%)
Severely active	26 (6.8%)
***Ostomy (eg, colostomy, ileostomy):***	
Yes	39 (10.2%)
No	343 (89.8%)
***Prescribed steroids:***	
Yes	55 (14.4%)
No	327 (85.6%)
***Number of IBD flare ups over the past year:***	
None	67 (17.5%)
1	78 (20.4%)
2	67 (17.5%)
3	52 (13.6%)
4	18 (4.7%)
5	22 (5.8%)
More than 5	78 (20.4%)
***Number of IBD related hospital admissions over the past year:***	
None	265 (69.4%)
1	67 (17.5%)
2	26 (6.8%)
3	9 (2.4%)
4	3 (0.8%)
5	3 (0.8%)
More than 5	9 (2.4%)

### 3.3. Covid-19 characteristics of study sample

The final questionnaire asked participants about their Covid-19 experiences. A full breakdown of responses can be seen in Table S1 in the Supplementary Material. Most respondents had not contracted Covid-19 (87.7%), while 3 participants tested positive, and 11.5% suspected they had it without formal testing. A total of 113 participants (29.6%) reported symptoms like cough, sore throat, fatigue, and high temperature. On a scale of 1–10, the mean belief about contracting the virus was 5.24 (*SD* = 1.91), indicating a perceived 50:50 chance. Regarding worry about contracting Covid-19 over the past month, 29 respondents (8.7%) had no worries, 188 (56.1%) occasionally worried, 84 (25.1%) worried much of the time, 24 (7.2%) worried most of the time, and 10 (3.0%) worried all the time.

### 3.4. Preliminary analyses of study outcomes and psychological inflexibility

Mean values and standard deviations for all outcome measures are displayed in Table S2 in the Supplementary Material. Participants were classified as having “low psychological inflexibility” if their AAQ-II score was lower than the mean (*M* = 24.16), and “high psychological inflexibility” if higher. Independent-samples t-tests showed significant differences between groups for all study variables. High psychological inflexibility participants had lower committed action (CAQ-8: *M = *24.97, *SD = *6.54) compared to low psychological inflexibility participants (*M = *31.97, *SD = *6.79) and this difference was significant, *t*(380)* = *-10.23, *P* < .001. They also had poorer IBD health-related quality of life (CUCQ-8: *M = *3.32, *SD = *1.46 vs. *M = *2.35, *SD = *1.36) which again was significant, *t*(380)* = *6.65, *P* < .001. There was a significant difference observed between the groups on total scores of the ISMI-29, *t*(380)* = *13.67, *P < *.001 with high psychological inflexibility participants showing higher internalized stigma (ISMI-29: *M = *2.23, *SD = *0.49 vs. *M = *1.60, *SD = *0.41) and on its subscales. High psychological inflexibility participants had higher psychological distress (DASS-21: *M = *57.17, *SD = *25.14 vs. *M = *25.97, *SD = *15.65) and on its subscales with differences between the group being statistically significant, *t*(295.85)* = *14.55, *P < *.001. High psychological inflexibility participants also had poorer IBD self-efficacy (IBDSES: *M = *154.54, *SD = *40.88 vs. *M = *197.66, *SD = *41.15, *t*(380)* = *−10.26, *P < *.001) and higher fatigue (*M = *20.62, *SD = *6.22 vs. *M = *16.71, *SD = *5.48, *t*(380)* = *6.53, *P < *.001). High psychological inflexibility participants scored higher on beliefs about emotions (BES: *M = *47.02, *SD = *13.68 vs. *M = *34.14, *SD = *13.55, *t*(380)* = *9.23, *P < *.001), and self-concealment (*M = *33.35, *SD = *9.88 vs. *M = *26.33, *SD = *9.43, *t*(380)* = *2.57.10, *P < *.001).

### 3.5. Prevalence of psychological distress

In the current study, psychological distress was measured using the DASS-21. Table 3 below provides the prevalence of psychological distress for the study sample relating to depression, anxiety, and stress based on the corresponding subscale scores of the DASS-21.

**Table 3.** Prevalence of psychological distress within the study sample.

DASS-21 scores	*n* (%)
***Depression Subscale***	
Minimal Symptoms	112 (29.3%)
Mild Symptoms	57 (14.9%)
Moderate Symptoms	97 (25.4%)
Severe Symptoms	59 (15.4%)
Extremely Severe Symptoms	57 (14.9%)
***Anxiety Subscale***	
Minimal Symptoms	211 (55.2%)
Mild Symptoms	25 (6.5%)
Moderate Symptoms	55 (14.4%)
Severe Symptoms	24 (6.3%)
Extremely Severe Symptoms	67 (17.5%)
***Stress Subscale***	
Minimal Symptoms	195 (51.0%)
Mild Symptoms	69 (18.1%)
Moderate Symptoms	45 (11.8%)
Severe Symptoms	47 (12.3%)
Extremely Severe Symptoms	26 (6.8%)

### 3.6. Prevalence of internalized stigma

In the current study sample, internalized stigma related to IBD was present for 40.5% of participants (27.2% mild, 10.2% moderate, and 3.1% severe) based on the total internalized stigma score. With regards to stigma resistance, 63.4% of the sample demonstrated high stigma resistance. Table 4 displays the percentage of minimal, mild, moderate, and severe stigma within the sample based on the ISMI-29 subscales.

**Table 4.** Prevalence of internalized stigma within the study sample.

	Minimal (%)	Mild (%)	Moderate (%)	Severe (%)
**Total Internalized Stigma**	59.6	27.2	10.2	3.1
Alienation	37.2	25.4	22.0	15.4
Stereotype Endorsement	91.4	6.0	1.6	1.0
Discrimination Experience	66.5	17.5	12.0	3.9
Social Withdrawal	54.2	22.5	14.4	8.9
	**Minimal (%)**	**Mild (%)**	**Moderate (%)**	**High (%)**
**Stigma Resistance**	2.4	6.8	27.5	63.4

Mean values and standard deviations for all outcome measures as a breakdown by level of internalized stigma are displayed in Table 5.

**Table 5.** Summary of outcome measures for study sample and comparison of mean scores based on level of internalized stigma.

			Level of internalized stigma			
**Variable**	**Categories**	**Total sample** **(*n* = 382)** ***M* (SD)**	**Minimal to none** **(*n* = 227)** ***M* (SD)**	**Mild** **(*n* = 104)** ***M* (SD)**	**Moderate** **(*n* = 39)** ***M* (SD)**	**Severe** **(*n* = 12)** ***M* (SD)**
Psychological Inflexibility (AAQ-II)	-	24.16 (9.61)	19.89 (7.80)	27.57 (7.60)	34.69 (7.14)	41.25 (5.55)
Committed Action (CAQ-8)	-	28.65 (7.52)	31.03 (6.85)	26.72 (7.25)	23.64 (4.61)	16.67 (5.61)
IBD HRQoL(CUCQ-8)	-	2.81 (1.49)	2.38 (1.30)	3.19 (1.46)	3.68 (1.48)	4.83 (1.48)
Internalised Stigma (ISMI-29)	Alienation	2.33 (0.73)	1.90 (0.52)	2.74 (0.37)	3.29 (0.45)	3.75 (0.25)
	Stereotype endorsement	1.45 (0.43)	1.21 (0.21)	1.65 (0.28)	1.95 (0.36)	2.68 (0.58)
	Discrimination experience	1.86 (0.63)	1.50 (0.42)	2.20 (0.37)	2.70 (0.44)	3.10 (0.38)
	Social withdrawal	2.03 (0.73)	1.56 (0.42)	2.49 (0.34)	3.08 (0.38)	3.62 (0.35)
	Stigma resistance	3.22 (0.48)	3.40 (0.38)	3.08 (0.41)	2.79 (0.44)	2.35 (0.67)
	Total scores	1.90 (0.55)	1.53 (0.29)	2.24 (0.13)	2.72 (0.14)	3.27 (0.14)
Psychological Distress (DASS-21)	Depression	16.15 (10.54)	12.03 (8.40)	19.29 (9.27)	26.05 (9.77)	34.67 (9.85)
	Anxiety	9.64 (9.79)	6.05 (6.89)	12.10 (9.66)	18.26 (11.00)	28.17 (9.78)
	Stress	15.87 (9.64)	12.60 (7.93)	18.71 (9.33)	22.72 (9.03)	31.00 (10.70)
	Total scores	41.66 (27.33)	30.68 (20.31)	50.10 (25.02)	67.03 (26.48)	93.83 (26.43)
Self-efficacy (IBD-SES)	-	177.23 (27.33)	195.70 (38.80)	157.01 (42.24)	139.87 (37.41)	124.33 (53.40)
Fatigue (CFS)	-	18.56 (6.15)	17.07 (5.50)	20.19 (6.25)	21.18 (7.09)	24.08 (4.74)
Beliefs About Emotions (BES)	-	40.24 (15.04)	34.27 (13.88)	46.77 (12.06)	52.26 (10.58)	57.58 (12.15)
Self-concealment (SCS)	-	29.66 (10.25)	26.66 (9.49)	32.67 (9.39)	34.90 (10.06)	43.33 (6.50)

One-way ANOVAs were conducted to analyze differences in mean scores between four internalized stigma groups for each study variable. Significant differences were observed between stigma groups on AAQ-II scores, *F*(3,378)* = *75.61, *P* < .001. Tukey post hoc tests revealed statistically significant differences in psychological inflexibility scores between all stigma groups. Significant differences were also found in CAQ-8 scores, *F*(3,378)* = *32.08, *P* < .001, with Tukey tests showing significant differences in committed action scores between all groups except “mild” and “moderate” stigma groups (*P* = .73). CUCQ-8 scores also showed significant differences, *F*(3,378)* = *24.39, *P* < .001, with Tukey tests indicating significant differences in IBD health-related quality of life scores between all groups except ‘mild’ and ‘moderate’ stigma groups (*P* = .73).

Significant differences were observed in DASS-21 Depression subscale scores, *F*(3,378)* = *54.73, *P* < .001, with Tukey tests showing significant differences between all stigma groups (*P* < .001). Anxiety subscale scores also showed significant differences, *F*(3,378)* = *51.22, *P* < .001, with Tukey tests indicating significant differences between all groups (*P* < .001). Stress subscale scores showed significant differences, *F*(3,378)* = *35.89, *P* < .001, with Tukey tests revealing significant differences between all groups except “mild” and “moderate” stigma groups (*P* = .62). Total DASS-21 scores showed significant differences, *F*(3,378)* = *60.72, *P* < .001, with Tukey tests indicating significant differences between all groups (*P* < .001).

IBDSES scores showed significant differences, *F*(3,378)* = *43.06, *P* < .001, with Tukey tests revealing significant differences between all groups except “mild” and “moderate” stigma groups (*P* = .11). CFS scores showed significant differences, *F*(3,378)* = *13.68, *P* < .001, with Tukey tests indicating significant differences between “minimal to no internalized stigma” group and other groups (*P* < .001), but not between other groups (*P* > .05).

BES scores showed significant differences, *F*(3,378)* = *42.57, *P* < .001, with Tukey tests revealing significant differences between “minimal to no internalized stigma” group and other groups (*P* < .001), and between “mild” and “severe” stigma groups (*P* = .04), but not between “moderate” and “severe” groups (*P* = .60). SCS scores showed significant differences, *F*(3,378)* = *23.55, *P* < .001, with Tukey tests indicating significant differences between all groups except “mild” and “moderate” stigma groups (*P* = .59).

### 3.7. Correlations between study variables

Bivariate correlation analyses revealed several significant relationships among study variables. Psychological inflexibility was negatively correlated with committed action (*r*(380) *=* −.60, *P* < .01), stigma resistance (*r*(380) *=* −.46, *P* < .01), and IBD self-efficacy (*r*(380) *=* −.59, *P* < .01). It was positively correlated with internalized stigma (*r*(380)* = *.68, *P* < .01), beliefs about emotions (*r*(380)* = *.52, *P* < .01), self-concealment (*r*(380)* = *.42, *P* < .01), decreased health-related quality of life (*r*(380)* = *.41, *P* < .01), fatigue (*r*(380)* = *.39, *P* < .01), and psychological distress (*r*(380)* = *.73, *P* < .01).

Internalized stigma was positively correlated with poorer health-related quality of life (*r*(380)* = *.43, *p < *.001), psychological distress (*r*(380)* = *.60, *P* < .01), beliefs about emotions (*r*(380)* = *.57, *P* < .01), and self-concealment (r(380)* = *.45, *P* < .01). It was negatively correlated with stigma resistance (*r*(380) *=* −.58, *P* < .01), IBD self-efficacy (*r*(380)* = *−57, *P* < .01), and committed action (*r*(380)* = *−48, *P < *.001). A weak positive correlation was found between internalized stigma and fatigue (*r*(380)* = *.36, *P* < .01). Correlations among all the study variables are presented in Table 6.

**Table 6.** Summary of correlations among study variables.

	1 *r* (*P*)	2 *r* (*P*)	3 *r* (*P*)	4 *r* (*P*)	5 *r* (*P*)	6 *r* (*P*)	7 *r* (*P*)	8 *r* (*P*)	9 *r* (*P*)	10 *r* (*P*)
**1. Psychological Inflexibility (AAQ-II)**	--									
**2. Committed Action** **(CAQ-8)**	−.60^**^	--								
**3. IBD HRQoL** **(CUCQ-8)**	.41^**^	−.21^**^	--							
**4. Internalised Stigma** **(ISMI-29)**	.68^**^	−.48^**^	.43^**^	--						
**5. Stigma Resistance** **(ISMI-29)**	−.46^**^	.45^**^	−.29^**^	−.58^**^	--					
**6. Psychological Distress (DASS-21)**	.73^**^	−.46^**^	.60^**^	.61^**^	−.43^**^	--				
**7. Self-efficacy** **(IBD-SES)**	−.59^**^	.45^**^	−.54^**^	−.57^**^	.54^**^	−.62^**^	--			
**8. Fatigue** **(CFS)**	.39^**^	−.27^**^	.52^**^	.36^**^	−.25^**^	.57^**^	−.45^**^	--		
**9. Beliefs About Emotions (BES)**	.52^**^	−.37^**^	.29^**^	.57^**^	−.41^**^	.48^**^	−.47^**^	.24^**^	--	
**10. Self-concealment** **(SCS)**	.44^**^	−.34^**^	.19^**^	.45^**^	−.37^**^	.39^**^	−.41^**^	.21^**^	.44^**^	--

**P < .01

Sociodemographic variables showed weak correlations with psychological inflexibility and internalized stigma. Age was negatively correlated with psychological inflexibility (*r*(380) *=* −.17, *P* < .01), and education level was negatively correlated with both psychological inflexibility (*r*(380) *=* −.32, *P* < .01) and internalized stigma (*r*(380) *=* −.33, *P* < .01). IBD severity over the past 3 months was positively correlated with both psychological inflexibility (*r*(380)* = *.194, *P* < .01) and internalized stigma (*r*(380)* = *.194, *P* < .01).

A point-biserial correlation indicated that psychological inflexibility was higher for individuals with an ostomy (*r*_pb_ (380)*=*-.11, *P = *.028) and those currently self-isolating due to Covid-19 (*r*_pb_ (380)* = *.11, *P = *.031). Internalized stigma was higher for individuals with an ostomy (*r*_pb_ (380) *=* −.14, *P = *.006) and those currently taking steroids (*r*_pb_ (380) *=* −.14, *P = *.006), as well as those self-isolating due to Covid-19 (*r*_pb_ (380)* = *.15, *P = *.003).

Demographics, such as ethnicity, employment status, relationship status, recent diagnosis, IBD diagnosis, and study recruitment source, were not associated with psychological inflexibility or internalized stigma. Variables like age, education level, presence of an ostomy, steroid prescription, IBD severity, and Covid-19 self-isolation status were included as covariates in the subsequent mediation analyses.

### 3.8. Mediation analyses

Psychological inflexibility was found to partially mediate the relationship between internalized stigma and a number of patient outcomes including psychological distress, IBD health-related quality of life, IBD self-efficacy, and self-concealment.

In all mediation models (see Figure S3 in the Supplementary Material) internalized stigma was associated with psychological inflexibility (*β* = .57, *P < *.001). Higher internalized stigma significantly predicted higher psychological distress (*β* = .63, *P < *.001). When controlling for internalized stigma, higher psychological inflexibility predicted higher psychological distress (*β* = .57, *P < *.001). Approximately 59% of the variance in psychological distress was explained by internalized stigma and psychological inflexibility, *F* (8,373) = 67.22, *P* < .001. A significant bootstrapped indirect effect was shown (*β* = .36, *SE* = .04; CI = .29–.44).

Higher internalized stigma significantly predicted lower IBD health-related quality of life (*β* = .20, *P < *.001). When controlling for internalized stigma, higher psychological inflexibility predicted lower IBD health-related quality of life (*β* = .11, *p* < .001). Approximately 53% of the variance in IBD health-related quality of life was explained by internalized stigma and psychological inflexibility, *F* (8,373) = 54.09, *p* < .001. A significant bootstrapped indirect effect was shown (*β* = .13, *SE* = .03; CI = .07–.19).

Higher internalized stigma significantly predicted lower IBD self-efficacy (*β* = −.37, *P < *.001). When controlling for internalized stigma, higher psychological inflexibility predicted lower IBD self-efficacy (*β* = −.31, *P* < .001). Approximately 45% of the variance in IBD self-efficacy was explained by internalized stigma and psychological inflexibility, *F* (8,373) = 39.30, *P* < .001. A significant bootstrapped indirect effect was shown (*β* = −.23, *SE* = .04; CI = −.31 to −.16).

Higher internalized stigma significantly predicted higher self-concealment (*β* = .24, *P < *.001). When controlling for internalized stigma, higher psychological inflexibility predicted higher self-concealment (*β* = .28, *P* < .001). Approximately 26% of the variance in self-concealment was explained by internalized stigma and psychological inflexibility, *F* (8,373) = 16.52, *P* < .001. A significant bootstrapped indirect effect was shown (*β* = .15, *SE* = .04; CI = .07–.23).

Higher internalized stigma significantly predicted higher levels of fatigue (*β* = .25, *P < *.001). The estimated effect of internalized stigma on fatigue, while controlling for psychological inflexibility was not significant (*β* = .11, *P* = .077). Approximately 23% of the variance in fatigue was explained by internalized stigma and psychological inflexibility, *F* (8,373) = 14.43, *P* < .001. A significant bootstrapped indirect effect was shown (*β* = .16, *SE* = .04; CI = .08–.25).

As shown in Figure S4 in the Supplementary Material, perceived stigma was associated with psychological inflexibility (*β* = .48, *P < *.001). Higher perceived stigma significantly predicted higher levels of internalized stigma (*β* = .68, *P* < .001). The estimated effect of perceived stigma on internalized stigma, while controlling for psychological inflexibility not significant (*β* = .50, *P* < .001). When controlling for psychological inflexibility, higher perceived stigma predicted internalized stigma (*β* = −.31, *P* < .001). Approximately 65% of the variance in internalized stigma was explained by perceived stigma and psychological inflexibility, *F* (8,373) = 88.50, *P* < .001. A significant bootstrapped indirect effect was shown (*β* = .18, *SE* = .02; CI = .14–.23).

## 4. Discussion

### 4.1. Summary of main findings

The study aimed to determine levels of internalized stigma, psychological inflexibility, health-related quality of life, psychological distress, IBD self-efficacy, fatigue, self-concealment, and beliefs about emotions among IBD adults. It also examined relationships among these variables and investigated the potential mediating effect of psychological inflexibility on the relationship between internalized stigma and key patient outcomes, as well as whether psychological inflexibility would mediate an association between public stigma and internalized stigma.

Internalised stigma related to IBD was present in 40.5% of participants, (27.2% mild, 10.2% moderate, 3.1% severe). Regarding stigma resistance, 63.4% demonstrated high resistance. These results align with Taft et al.,^60^ who found internalized stigma in 36% of their 191 participants (29% mild, 7% moderate, and 0% severe) and high stigma resistance in 46%.^60^ Consistent with Taft et al.’s findings, patient outcomes were poorer as the level of internalized stigma increased and in addition, individuals from lower educational backgrounds and with more active disease reported greater levels of internalized stigma, highlighting the need for healthcare professionals to consider these factors to improve patient outcomes. Longitudinal studies could explore how internalized stigma changes over time dependent on clinical presentation.

No published literature currently examines the relationship between psychological inflexibility, internalized stigma, and patient outcomes in adult IBD populations. The study hypothesized that individuals less susceptible to internalizing stigma would have lower psychological inflexibility, greater awareness of their internal experiences, and be more open to experiencing stigmatizing thoughts. These individuals would be better at labeling and defusing from stigmatizing thoughts, continuing to engage in valued actions despite their presence.

The study hypothesized that adults more susceptible to internalizing stigma would have higher psychological inflexibility and poorer patient outcomes, including increased psychological distress, poorer health-related quality of life, poorer self-efficacy, and increased fatigue. It also predicted that internalized stigma and psychological inflexibility would be associated with higher self-concealment and beliefs about the unacceptability of negative emotions. Psychological inflexibility was expected to mediate the associations between internalized stigma and patient outcomes. Study findings supported this hypothesis, showing that individuals with lower psychological inflexibility had lower levels of internalized stigma, higher committed action, better health-related quality of life, higher self-efficacy, lower self-concealment, lower fatigue, and fewer beliefs about the unacceptability of negative emotions.

Correlation analyses revealed significant associations between scores on the BES and other study variables. Individuals less accepting of negative emotions had higher psychological inflexibility, lower committed action, higher internalized stigma, higher psychological distress, higher self-concealment, higher fatigue, and poorer health-related quality of life. This aligns with previous research linking beliefs about negative emotions to poorer health outcomes in conditions like fibromyalgia^119^ and irritable bowel syndrome.^120^

The study’s findings on the relationship between psychological inflexibility and internalized stigma align with a recent meta-analytic review on psychological inflexibility and stigma.^121^ This review found significant correlations between psychological inflexibility and internalized stigma related to mental illness, epilepsy, internalized homophobia, substance use, and body image shame. The study also examined pathways by which internalized stigma may lead to poorer patient outcomes, finding that psychological inflexibility mediated these relationships while controlling for covariates (eg, age, level of education, presence of an ostomy, being prescribed steroids, IBD symptom severity over the past 3 months, and current Covid-19 isolation status).

Psychological inflexibility completely mediated the association between internalized stigma and fatigue, which fits with previous research suggesting a link between psychological inflexibility and energy levels.^122,123^ High energy levels may provide the resources needed to challenge negative internal experiences and engage in flexible responses. Ongoing stressors associated with IBD may disrupt energy levels and fatigue, linking IBD severity to psychological inflexibility and internalized stigma. Individuals experiencing discrimination regarding their IBD who cannot employ flexible responses may spend more energy avoiding stigmatizing experiences, making it harder to label and defuse from them and engage in meaningful activities.

The study also examined whether psychological inflexibility would mediate an association between public stigma (also referred to as perceived stigma or enacted stigma as measured by the Discrimination Experience subscale of the ISMI-29^114,115^) and internalized stigma. Internalised stigma develops through awareness of public stigma, agreement with negative assumptions, and applying those stereotypes to oneself.^124^ The model highlights the interconnectedness of public and internalized stigma, with 65% of the variance in internalized stigma explained by perceived stigma and psychological inflexibility. Individuals with lower psychological inflexibility would be more aware of their internal experiences and open to experiencing stigmatizing thoughts, making them less likely to internalize these thoughts. Emerging research indicates that familiarity with IBD can reduce public stigma^125,126^ and therefore, it may be important to highlight that organizations and healthcare providers may wish to consider IBD awareness campaigns to increase public understanding of IBD, thereby reducing stigma and enhancing the quality of life for individuals who are affected.

Our study’s findings suggest that lower internalized stigma is associated with increased psychological flexibility and better patient outcomes, indicating implications for clinical treatment. Acceptance and Commitment Therapy (ACT) aims to increase psychological flexibility by changing individuals’ relationships with their thoughts, feelings, and behaviors.^61^ ACT may lend itself well to individuals with internalized stigma related to their IBD by helping them to manage their distressing internal experiences (eg, stigmatizing thoughts) more effectively so that they can engage in a rich, meaningful life regardless of their IBD diagnosis. Research from social psychology suggests that attempts to control prejudiced thoughts can backfire,^127,128^ making ACT’s approach of observing internal experiences without trying to change them potentially beneficial.

The use of ACT as a therapeutic intervention in IBD populations is still in its early stages, with only three published studies to date. One RCT showed improvements in stress coping strategies,^129^ another study showed significant decreases in anxiety scores,^130^ and a third RCT showed reductions in stress and depression scores and increased psychological flexibility.^131^ ACT has been well-received in other chronic health conditions by health-care professionals and patients alike^132^ and is emerging in gastroenterology.^133^ Although some ACT studies have indicated reductions in stigma in other populations,^121^ no studies have explicitly examined ACT interventions to reduce IBD-related stigma. The study’s findings highlight the importance of psychological inflexibility in the relationship between internalized stigma and patient outcomes, warranting the need to evaluate ACT interventions aiming to increase psychological flexibility and target stigma within IBD populations.

### 4.2. Limitations of the current study

There are a number of limitations of the current study which should be highlighted when considering the conclusions that can be drawn from its findings. First, it uses a cross-sectional quantitative design, limiting the ability to infer directional relationships between variables. Longitudinal data would be needed to confirm the directionality of mediation analyses. Furthermore, longitudinal studies could explore how internalized stigma may change over time dependent on patients’ clinical presentation. Future studies should also examine psychological flexibility, internalized stigma, and patient outcomes over multiple time points, such as pre-and-post an ACT intervention.

Second, participants provided data via self-report measures, which may be subject to response bias. Online recruitment could also introduce bias, as the sample was self-selecting and likely engaged with IBD charities or content online. This may exclude individuals with more internalized stigma who avoid IBD-related content. However, the prevalence of internalized stigma in this sample was comparable to a previous study.^60^

Another limitation is the inability to confirm participants’ IBD diagnoses. The survey required participants to confirm their diagnosis, but this could not be verified. The study was initially planned to recruit from outpatient clinics, but due to the Covid-19 pandemic, it was modified to recruit solely online. Previous research indicates that online-recruited participants may report poorer health-related quality of life compared to those recruited in clinics^134^ and as such our sample may not be as representative of those with IBD in the general population.

A further limitation arises as similarly to Taft et al.’s study^60^ we used a modified version of the ISMI-29 to measure internalized stigma in an IBD population. Although reliability statistics were acceptable, ideally the ISMI-29 should be validated with IBD to ensure that stigma constructs are being measured successfully. The AAQ-II and CAQ-8 were used to measure psychological inflexibility and committed action, respectively. We primarily stuck to using the AAQ-II as this appears to be the most widely used measure of psychological inflexibility^66^ and hence would lower participant burden, but other facets of psychological flexibility, such as mindfulness, were not explored. It also needs to be acknowledged that given the relative novelty of the Covid-19 pandemic at the time when our study began recruitment, no specific measures relating to experiences of the pandemic were available for use, and as such the Covid-19 questionnaire was a bespoke measure and not validated, potentially missing relevant aspects of participants’ experiences which could have been relevant.

Finally, it should be noted that our sample was predominantly female (*n = *302; 79.1%), and this may mean that the experiences of men with IBD are not captured accurately in our findings. Research shows variations in incidence, prevalence, clinical symptoms, comorbidities, and disease severity observed between men and women across age groups suggesting significant sex-based differences in IBD.^135,136^ As such the clear gender bias in our sample might impact our findings or limit generalisability to the target population.

Additionally, the vast majority of our sample was White/Caucasian (*n = *334; 87.4%). While this is reflective IBD being more predominant within the white population,^137^ caution should be used when applying our findings to other ethnic groups for whom experiences of internalized stigma and IBD-related outcomes may differ. It could be argued that the cultural and social diversity of different regions may not be fully represented in our sample. Expanding the demographic scope in future research could help provide a more comprehensive understanding of these factors.

Also of note, the distribution of CD and UC diagnoses in our sample differs from expected prevalence rates,^2,4^ with CD being overrepresented (45% vs. 33–40%), UC underrepresented (42.9% vs. 60–67%), and IBD-unclassified slightly higher than usual (12% vs. 5–10%). As such, our sample provides insights into the IBD population, but it may not be fully representative of the general IBD population due to deviations in the proportions of CD, UC, and IBD-unclassified cases compared to expected epidemiological distributions.

Despite these limitations relating to our sample, it should be highlighted that we did manage to recruit a large number of participants (*n = *382) and that the mean age of our sample fits with research of our target population which highlights the highest incidence of IBD is observed between the second and fourth decades of life.^138^ Our sample is also similar to other cross-sectional survey studies using IBD populations (eg,^60,139–141^), in regards to gender, age, ethnicity, and severity of disease symptoms. However, it is important to note that individuals experiencing more severe disease symptoms or active flares may have been underrepresented in the current study due to the additional burden of participation.

### 4.3. Conclusions

To our knowledge, this study is the first to examine the relationship between psychological inflexibility, internalized stigma, and patient outcomes in adults with IBD. This study’s findings extended previous research looking at internalized stigma within this population by examining the mediating role of psychological flexibility. Findings suggest that interventions like ACT, which aim to increase psychological flexibility, could improve quality of life for individuals with IBD and internalized stigma by helping them manage distressing thoughts. Future research should explore changes in internalized stigma over time or implement and evaluate pre-and-post ACT intervention outcomes for patients with IBD who experience internalized stigma.

## Supplementary Material

jjaf055_suppl_Supplementary_Materials

## Data Availability

The data underlying this article will be shared on reasonable request to the corresponding author.
