# Supplementary material for: The mediating role of psychological inflexibility on internalized stigma and patient outcomes in a sample of adults with inflammatory bowel disease
Source: J Crohns Colitis. 2025 Apr 1;19(5):jjaf055. doi: 10.1093/ecco-jcc/jjaf055 (PMC12060869; doi:10.1093/ecco-jcc/jjaf055)
Supplement: jjaf055_suppl_Supplementary_Materials [file jjaf055_suppl_supplementary_materials.docx]

**Supplementary Material**

**Acceptance**

(Experiential

Avoidance)

**Contact with the**

**present moment**

(conceptualised past

and feared future)

**Values**

(lack of values

clarity)

**Committed**

**Action**

(Inaction,

impulsivity,

or avoidance)

**Cognitive**

**Defusion**

(Fusion)

**Self as Context**

(Attachment to

conceptualised self)

**Psychological Flexibility**

(Psychological Inflexibility)

***Figure S1 - The Hexaflex model of ACT illustrating the core processes underlying ‘psychological flexibility’ with processes in parentheses illustrating ‘psychological inflexibility’.***

**X**

**M**

**Y**

*a*

*b*

*‘c*

**X**

**Y**

*c*

***Figure S2 – Simple mediation model. Path diagrams show the total effect (c) of independent variable (X) on dependent variable (Y) and the indirect effect (‘c) of independent variable (X) on the dependant variable (Y) through the mediating variable (M).***

**Internalised Stigma**

**Psychological Inflexibility**

**Health Related Quality of Life†**

*.63****

.20***

*.24*** (.11***)*

**Internalised Stigma**

**Psychological Inflexibility**

**Psychological Distress**

*.63****

.57***

*. 53*** (.17***)*

***(† - Increased scores on CUCQ-8 measure are indicative of poorer health related quality of life).***

**Internalised Stigma**

**Psychological Inflexibility**

**IBD Self Efficacy**

*.63****

-.37***

*-.55*** (-.31***)*

**Internalised Stigma**

**Psychological Inflexibility**

**Self-Concealment**

*.63****

.24***

*.43*** (.28***)*

**Internalised Stigma**

**Psychological Inflexibility**

**Fatigue**

*.63****

.25***

*.27*** (.11)*

****p < .001*

***Figure S3 – Models summarising psychological inflexibility as a mediator between internalised stigma and several patient outcomes. Standardised regression coefficients are displayed. The standardised regression coefficient between internalised stigma and each patient outcome, controlling for psychological inflexibility, is shown in parentheses.***

**Perceived Stigma**

**Psychological Inflexibility**

**Internalised Stigma**

*.48****

.39***

*.68*** (.50***)*

****p < .001*

***Figure S4 - Model summarising psychological inflexibility as a mediator between perceived stigma (discrimination experience subscale of ISMI-29) and internalised stigma. Standardised regression coefficients are displayed. The standardised regression coefficient between perceived stigma and internalised stigma, controlling for psychological inflexibility, is shown in parentheses.***

| **Variable** | **n (%)** |
| --- | --- |
| ***Have you had Covid-19 (coronavirus)?*** |  |
| Yes, diagnosed and recovered | 1 (0.3%) |
| Yes, diagnosed and still ill | 2 (0.5%) |
| Suspected, and recovered | 40 (10.5%) |
| Suspected, and still ill | 4 (1.0%) |
| No | 335 (87.7%) |
| ***What do you believe your risk of getting Covid-19 is on a scale of 1 - 10?*** |  |
| 1 - Extremely unlikely | 11 (3.3%) |
| 2 - Very unlikely | 21 (6.3%) |
| 3 - Unlikely | 25 (7.5%) |
| 4 - Somewhat unlikely | 37 (11%) |
| 5 - 50:50 chance | 118 (35.2%) |
| 6 - Somewhat likely | 27 (8.1%) |
| 7 - Likely | 58 (17.3%) |
| 8 - Very likely | 22 (6.6%) |
| 9 - Extremely likely | 13 (3.9%) |
| 10 - I will definitely get Covid-19 | 3 (0.9%) |
| ***Over the past month:*** |  |
| I have not worried at all about getting Covid-19 | 29 (8.7%) |
| I occasionally worried about getting Covid-19 | 188 (56.1%) |
| I spent much of my time worrying about getting Covid-19 | 84 (25.1%) |
| I spent most of my time worrying about getting Covid-19 | 24 (7.2%) |
| I spent all of my time worrying about getting Covid-19 | 10 (3.0%) |
| ***Experienced Covid-19 symptoms?*** |  |
| Yes | 113 (29.6%) |
| No | 269 (70.4%) |
| ***Life events experienced as a direct result of the Covid-19 pandemic:*** |  |
| Death of a spouse / partner | 0 (0%) |
| Separation/divorce | 6 (1.6%) |
| Being sacked at work/losing my job | 22 (5.8%) |
| Death of a close friend/family member | 21 (5.5%) |
| Worsening of personal finances | 96 (25.1%) |
| Worsening of living conditions | 39 (10.2%) |
| None of the above have happened to me | 242 (63.4%) |
| ***Living situation:*** |  |
| Living with others | 325 (85.1%) |
| Living alone | 57 (14.9%) |
| ***Living with someone who has had Covid-19 (if applicable):*** |  |
| Yes | 7 (2.2%) |
| Suspected | 35 (10.8%) |
| No | 282 (87%) |
| ***Current Isolation Status:*** |  |
| Not self-isolating | 183 (47.9%) |
| Self-isolating | 199 (52.1%) |

***Table S1 – Covid-19 characteristics of study sample***

| **Outcome Measure** | **Categories** | **Total Sample**  **(n = 382)**  ***M* (*SD*)** | **Low Psychological Inflexibility Group**  **(n = 201)**  ***M* (*SD*)** | **High Psychological Inflexibility Group**  **(n = 181)**  ***M* (*SD*)** | ***p v*alue** |
| --- | --- | --- | --- | --- | --- |
| Psychological Inflexibility (AAQ-II) | - | 24.16 (9.61) | 16.70 (5.11) | 32.44 (5.94) | *** |
| Committed Action (CAQ-8) | - | 28.65 (7.52) | 31.97 (6.79) | 24.97 (6.54) | *** |
| Health-related QoL (CUCQ-8) | - | 2.81 (1.49) | 2.35 (1.36) | 3.32 (1.46) | *** |
| Internalised Stigma  (ISMI-29) | Alienation | 2.33 (0.73) | 1.94 (0.59) | 2.76 (0.62) | *** |
|  | Stereotype endorsement | 1.45 (0.43) | 1.27 (0.29) | 1.66 (0.47) | *** |
|  | Discrimination experience | 1.86 (0.63) | 1.59 (0.52) | 2.17 (0.61) | *** |
|  | Social withdrawal | 2.03 (0.73) | 1.67 (0.56) | 2.43 (0.69) | *** |
|  | Stigma resistance | 3.22 (0.48) | 3.40 (0.39) | 3.03 (0.50) | *** |
|  | Total scores | 1.90 (0.55) | 1.60 (0.41) | 2.23 (0.49) | *** |
| Psychological Distress  (DASS-21) | Depression | 16.15 (10.54) | 10.60 (6.90) | 22.72 (10.19) | *** |
|  | Anxiety | 9.64 (9.79) | 4.39 (4.95) | 13.15 (9.00) | *** |
|  | Stress | 15.87 (9.64) | 10.99 (6.75) | 21.30 (9.47) | *** |
|  | Total scores | 41.66 (27.33) | 25.97 (15.65) | 57.17 (25.14) | *** |
| Self-efficacy (IBD-SES) | - | 177.23 (27.33) | 197.66 (41.15) | 154.54 (40.88) | *** |
| Fatigue (CFS) | - | 18.56 (6.15) | 16.71 (5.48) | 20.62 (6.22) | *** |
| Beliefs About Emotions (BES) | - | 40.24 (15.04) | 34.14 (13.55) | 47.02 (13.68) | *** |
| Self-concealment (SCS) | - | 29.66 (10.25) | 26.33 (9.43) | 33.35 (9.88) | *** |

**** p <0.001*

***Table S2 – Summary of outcome measures for study sample and comparison of mean scores based on level of psychological flexibility***

***File S1 - Socio-Demographic & IBD Status Questionnaire***

Please answer the questions below about you and your IBD.

**1. How old are you?** **Please state in years below.**

________ years

**2. Which gender do you identify with? Please circle one of the following options.**

- 1. Male
  2. Female
  3. Other
  4. Prefer not to say

**3. How would you describe your ethnic group? Please circle one of the following options.**

| **WHITE** | **ASIAN / ASIAN BRITISH** |
| --- | --- |
| English / Scottish / Welsh / Northern Irish / Irish | Indian |
| Gypsy or Traveller | Pakistani |
| Other White | Bangladeshi |
| **MIXED / MULTIPLE ETHNIC GROUPS** | Chinese |
| White and Black Carribbean | Other Asian / Asian British |
| White and Black African | **BLACK / AFRICAN / CARRIBEAN / BLACK BRITISH** |
| White and Asian | Carribean |
| Other Mixed / Multiple ethnic groups | African |
|  | Other Black / African / Carribbean / Black British |
| **OTHER ETHNIC GROUP** | |
| Arab | |
| Other Ethnic Group | |

**5. What is your current employment status? Please circle one of the options.**

- 1. Employed full-time
  2. Employed part-time
  3. Self-employed
  4. Unemployed (currently looking for work)
  5. Unemployed (not currently looking for work)
  6. Student
  7. Retired
  8. Homemaker
  9. Unable to work.

**6. What is your current relationship status**

- 1. Single
  2. Married
  3. Widowed
  4. Separated
  5. Co-habiting
  6. Long Term
  7. Civil Partnership
  8. Divorced
  9. Prefer not to say

**7. What is the highest educational qualification you have completed?**

- 1. No qualifications
  2. Foundation diploma / GCSE (grades D-G) / NVQ Level 1
  3. Higher diploma / GCSE (grades A* - C) / NVQ Level 2
  4. Advanced diploma / A Level / BTEC National / NVQ Level 3
  5. Certificate of Higher Education / BTEC Professional / NVQ Level 4
  6. Bachelor’s degree (with honours)
  7. *Postgraduate certificate or diploma / Master’s degree*
  8. *Doctorate degree*

***8. Which of these best describes yours IBD diagnosis?***

1. I have Crohn’s Disease
2. I have Ulcerative Colitis
3. I have Microscopic Colitis
4. I have Inflammatory Bowel Disease Unclassified
5. I have symptoms but a hospital specialist has yet to confirm that I have IBD*
6. I do not have Chron’s or Colitis*

** trigger end of survey*

***9. How recently were you diagnosed with Crohn’s or Colitis?***

*Note: Sometimes a patient’s diagnosis may change (although it is not a common occurrence). If your diagnosis has changed please base your answers on your most recent confirmed diagnosis.*

*A confirmed diagnosis is made within a specialist setting, usually at a hospital, using a combination of medical tests and investigations, including blood tests, scans (e.g. MRI, CT scan, barium studies or chest x-rays) and endoscopies (such as colonoscopy or a sigmoidoscopy, which uses a long flexible tube to take pictures and biopsies).*

1. Within the last 2 years
2. More than 2 years ago
3. Don’t know / can’t remember

If you know the exact amount of time you have been diagnosed, please indicate the length of time in years below:

________ years since most recent confirmed diagnosis

***10.*** **When was your last hospital contact regarding your IBD?**

- 1. Within the last 12 months
  2. 1 – 2 years ago
  3. More than 2 years ago
  4. Never had contact

***11.*** **How many flares have you experienced in the last 12 months?**

*Note: Please select ‘None’, if you have been recently diagnosed and have yet to get symptoms under more control or experience remission.*

*If you are not sure,* ***please give your best estimate.***

*A flare can be described in some of the following ways:*

- *Going to the toilet more than 5 times in 24 hours or more than is normal for you*
- *Loose stools or diarrhoea with any blood/mucus for more than 3 days*
- *Abdominal pain*

*Flares aren’t always just in the gut and can also include inflammatory symptoms outside the bowel getting worse such as, mouth ulcers, joint pain and skin rashes etc.*

a) None b) 1 c) 2 d) 3 e) 4 f) 5 g) More than 5

**12. How many times have you been admitted to hospital because of Crohn’s or Colitis-related symptoms in the last 12 months?**

a) None b) 1 c) 2 d) 3 e) 4 f) 5 g) More than 5

**13. How would you generally describe the severity of your Crohn’s or Colitis over the last 3 months?**

1. **Not active** – I have no symptoms and have not been affected by my disease
2. **Minimally active** – I have symptoms, but my symptoms have not got in the way of everyday activities
3. **Mildly active** – my symptoms have got in the way of some everyday activities
4. **Moderately active** – my symptoms have significantly got in the way of everyday activities
5. **Severely active** – I have been unable to perform everyday activities

**14. Are you currently dependent on steroid (corticosteroid) medication as a result of your IBD?**

a) Yes b) No

**15. Do you currently have an ostomy (e.g. colostomy, ileostomy)?**

a) Yes b) No

***File S2 – Covid-19 Questionnaire***

Coronavirus disease 2019 (Covid-19) is an infectious disease caused by severe acute respiratory syndrome coronavirus 2 (SARS-CoV-2). The disease was first identified in December 2019 in Wuhan, the capital of China's Hubei province, and has since spread globally, resulting in the ongoing 2019–2020 coronavirus pandemic.

We would like to ask you about the potential adjustments/impact the Covid-19 pandemic has had on your life.

**1a) Have you had Covid-19 (coronavirus)?**

a) Yes, diagnosed and recovered

b) Yes, diagnosed and still ill

c) Suspected, and recovered

d) Suspected, and still ill

e) No

**1b) If Yes: When were you tested for Covid-19?**

1. Past week
2. Past 2 weeks
3. Past month
4. More than 2 months ago
5. More than 6 months ago
6. More than a year ago
7. N/A

**2a) Have you experienced symptoms you think might be related to Covid-19?**

a) Yes b) No

**2b) If Yes: Which symptoms did you experience? (Please tick all that apply)**

 Persistent Cough

 Fatigue

 Shortness of breath

 Loss of smell

 Loss of taste

 Sore throat

 Tightness in chest

 Diarrhoea

 Loss of appetite

 Feeling confused

 Fever/High Temperature (More than 39°C/102.2°F)

 Other (please state)

_________________________

**2c) If Yes: How severe were your symptoms on a scale of 1-10?**

| 1  Very mild  symptoms | 2 | 3 | 4 | 5 | 6 | 7 | 8 | 9 | 10  Very severe / I was hospitalised |
| --- | --- | --- | --- | --- | --- | --- | --- | --- | --- |
|  |  |  |  |  |  |  |  |  |  |

**2d) If Yes: When did you experience these symptoms?**

1. Currently
2. Past 2 weeks
3. Last month
4. More than 2 months ago
5. More than 6 months ago
6. More than a year ago

**3) Are you currently working?**

1. Yes, I am going to work (working on site as usual)
2. Yes, I am working from home (working remotely)
3. No, I’m a student
4. No, I was unemployed before the Covid-19 pandemic
5. No, I was retired before the Covid-19 pandemic
6. No, I’m a homemaker
7. No, I am on sick leave
8. No, I lost my job as a result of the Covid-19 pandemic

**4) As a result of Covid-19 pandemic, have any of the following events happened to you? (Please tick all that apply):**

 Death of a spouse / partner

 Separation/divorce

 Being sacked at work/losing my job

 Death of a close friend/family member

 Worsening of personal finances

 Worsening of living conditions

 None of the above have happened to me

**5a) Do you currently live in the same household as someone else?**

a) Yes, I live with others b) No, I live alone

**5b) If Yes: How many others do you live with who are over the age of 18?**

___________

**5c) If Yes: How many other do you live with who are under the age of 18?**

___________

**5d) If Yes: Has anyone you live with had Covid-19?**

1. Yes b) Suspected c) No

**6a) Do you have any caring responsibilities for others who do not live with you?**

1. Yes b) No

**6b) If Yes: Has the person/people you care for who do not live in your household had Covid-19?**

1. Yes b) No

**7) What is your current self-isolation status?**

1. I am living my life as normal
2. I am NOT self-isolating, but I have cut down on my usual activities as a precaution/I am social distancing
3. I am NOT self-isolating specifically, but I have stopped going to work like normal and am working from home
4. I am self-isolating due to a diagnosis of Covid-19 or possible symptoms
5. I am self-isolating because I have an existing medical condition or am categorised as high-risk.
6. I am self-isolating as I am worried about spreading it to others or getting ill (but I am not high risk)
7. I am self-isolating to protect a family member, friend or housemate who has an existing medical condition/is high risk
8. I am self-isolating as it has been ordered by the government or local authority as part of a lockdown
9. I am self-isolating but this is NOT because of Covid-19 but because of another reason (e.g. a pre-existing health condition or disability that required me to self-isolate prior to Covid-19).

**8) How many times in the past two weeks have you left your house (e.g. to go food shopping or for exercise)? Please estimate if unsure.**

___________

**Beliefs about risk (skip if you have already tested positive for Covid-19)**

**9) On a scale of 0-10 what do you believe your risk of getting Covid-19 is?**

| 0 | 1 | 2 | 3 | 4 | 5 | 6 | 7 | 8 | 9 | 10 |
| --- | --- | --- | --- | --- | --- | --- | --- | --- | --- | --- |
| I definitely won’t get Covid-19 | Extremely unlikely | Very unlikely | Unlikely | Somewhat unlikely | 50:50 chance | Somewhat likely | Likely | Very likely | Extremely likely | I will definitely get Covid-19 |

**10) On a scale of 0 - 10 how severe do you think your symptoms would be if you did get Covid-19?**

| 0 | 1 | 2 | 3 | 4 | 5 | 6 | 7 | 8 | 9 | 10 |
| --- | --- | --- | --- | --- | --- | --- | --- | --- | --- | --- |
| I would not have any symptoms |  |  |  |  |  |  |  |  |  | I will likely die from Covid-19 |

**11) Please read each statement carefully and then select the one which best describes how you have felt OVER THE PAST MONTH. If you find that more than one statement applies, please select any of the applicable statements.**

 I have not worried about getting Covid-19.

 I occasionally worried about getting Covid 19.

 I spent much of my time worrying about getting Covid-19.

 I spent most of my time worrying about Covid-19.

 I spent all of my time worrying about Covid-19.
